# Supervision and autonomy of ophthalmology residents in the outpatient Clinic in the United States: a survey of ACGME-accredited programs

**DOI:** 10.1186/s12909-017-0941-0

**Published:** 2017-06-26

**Authors:** Eric L. Singman, Divya Srikumaran, Laura Green, Jing Tian, Peter McDonnell

**Affiliations:** 1 Wilmer Eye Institute General Eye Services Clinic, @ Johns Hopkins Hospital, Wilmer B-29, 600 N. Wolfe St, Baltimore, MD 21287 USA; 2 Ophthalmology Residency Program Director, Lifebridge Health Krieger Eye Institute, 2411 W. Belvedere Ave, Baltimore, MD 21215 USA; 3 Biostatistics Consulting Center, Johns Hopkins University Bloomberg School of Public Health, 615 N. Wolfe St, Room 3148, Baltimore, MD 21287 USA; 4 Wilmer Eye Institute, @ Johns Hopkins Hospital, Maumenee 727, 600 N. Wolfe St, Baltimore, MD 21287 USA

**Keywords:** Supervision, Autonomy, Resident, Ophthalmology, Education, Training, Outpatient, Benchmark

## Abstract

**Background:**

The development and demonstration of incremental trainee autonomy is required by the ACGME. However, there is scant published research concerning autonomy of ophthalmology residents in the outpatient clinic setting. This study explored the landscape of resident ophthalmology outpatient clinics in the United States.

**Methods:**

A link to an online survey using the QualtricsTM platform was emailed to the program directors of all 115 ACGME-accredited ophthalmology programs in the United States. Survey questions explored whether resident training programs hosted a continuity clinic where residents would see their own patients, and if so, the degree of faculty supervision provided therein. Metrics such as size of the resident program, number of faculty and clinic setting were also recorded. Correlations between the degree of faculty supervision and other metrics were explored.

**Results:**

The response rate was 94%; 69% of respondents indicated that their trainees hosted continuity clinics. Of those programs, 30% required a faculty member to see each patient treated by a resident, while 42% expected the faculty member to at least discuss (if not see) each patient. All programs expected some degree of faculty interaction based upon circumstances such as the level of training of the resident or complexity of the clinical situation. 67% of programs that tracked the contribution of the clinic to resident surgical caseloads reported that these clinics provided more than half of the resident surgical volumes. More ¾ of resident clinics were located in urban settings. The degree of faculty supervision did not correlate to any of the other metrics evaluated.

**Conclusions:**

The majority of ophthalmology resident training programs in the United States host a continuity clinic located in an urban environment where residents follow their own patients. Furthermore, most of these clinics require supervising faculty to review both the patients seen and the medical documentation created by the resident encounters. The different degrees of faculty supervision outlined by this survey might provide a useful guide presuming they can be correlated with validated metrics of educational quality. Finally, this study could provide an adjunctive resource to current international efforts to standardize ophthalmic residency education.

**Electronic supplementary material:**

The online version of this article (doi:10.1186/s12909-017-0941-0) contains supplementary material, which is available to authorized users.

## Background

The ultimate goal of a residency training program is to create competent and independent medical practitioners. Residents themselves consider autonomy to be important, whether in the operating room [1–5], the inpatient ward [6–9] or the outpatient setting [10, 11]. Furthermore, insufficient autonomy not only compromises readiness for graduation, but also has been reported to be a factor in resident burnout [12–14]. Currently there is a trend toward increased supervision of residents in response to the 2011 ACGME guidelines created to enhance patient care and safety and improve resident education [15]; by the same token, the ACGME also requires a demonstration of graduated autonomy for residents. Published reports suggest that autonomy may not be diminished by enhanced supervision in certain care settings such as overnight on-call [9, 16, 17]. In addition, residents have reported frequent instances in which they felt there was insufficient supervision [18]. However there is a significant concern that efforts are needed to ensure that a balance can be struck between supervision and autonomy so that patient safety and resident education are both maximized [19–21].

Ophthalmology is an outpatient specialty and most practicing ophthalmologists spend the majority of their time in the clinic. This setting, therefore, is where residents develop the core competencies as outlined by the ACGME [22]. Notably the preponderance of the literature on resident supervision and autonomy has been carried out in the inpatient and/or surgical setting, making extrapolation to outpatient specialties difficult or of questionable validity. Indeed, our computerized literature search (Pubmed) found that there are no published reports of ophthalmology resident training as it pertains to supervision and autonomy in the outpatient setting. The goal of this study is to increase awareness of this topic. Toward that end, our team created the Ophthalmology Program Directors’ Study Group (OPDSG) to survey ophthalmology residency program directors on their approach to resident-hosted continuity clinics. It is hoped that documenting how supervision and autonomy are negotiated in different training programs might help better understand best practices for education, particularly if the degree of supervision and autonomy can be compared to other measures of programmatic success. Such a measure might also provide a tool for programs to improve the balance of supervision and autonomy in their programs, if that balance is perceived to be inappropriate by residents based upon the annual ACGME survey.

## Methods

A 24-question online survey was created using the Qualtrics™ platform and emailed to all 115 ACGME-accredited ophthalmology residency program directors in February 2016. Follow-up emails and telephone calls by the authors were placed regularly to ensure that emails were correctly directed. In two cases, surveys were performed by telephone. Program coordinators were also offered the opportunity to respond when it seemed that the program directors might not. To encourage responses, a $10 charitable donation was pledged to Research to Prevent Blindness for every program involved in the study, totaling $1150. The initial email included an introductory letter explaining the purpose of the study and confirming that this study was acknowledged by the Johns Hopkins Institutional Review Board.

The survey was designed using forced choices, although in some instances, responders were permitted to provide additional free-text information. The survey uses simple Boolean logic so that responders could be directed to germane questions based upon their answers while skipping over questions that might be redundant or non-sequitur. The survey as it appeared to respondents can be accessed with this link: https://jhmi.co1.qualtrics.com/SE/?SID=SV_2tXMkHfd1SykEGp

The survey questions and the logic-flow instructions based upon the answers to each question are presented as Additional file 1. The survey specifically asked whether a program hosted a resident continuity clinic. The clinic was defined as an outpatient site where residents provide continuity care to their own comprehensive patients. Notably, whether a program provided this experience or not, demographics from each program were requested including environment in which the clinic stood, number of residents and number of core-, full-time and part-time faculty. In addition, all responders were offered the opportunity to participate in our new working group, dubbed the Ophthalmology Program Directors’ Study Group (OPDSG).

If respondents indicated that they did host a resident continuity clinic, they were asked how that clinic was supervised and the circumstances under which a supervisor might need to discuss the case, see a patient after the resident provides care, or review the resident documentation. In addition they were asked whether they had plans to discontinue the clinic and the associated reasoning, as well as whether they tracked the contribution to resident surgical volume provided through the clinic. For those programs that did not host a clinic, respondents were asked whether they were considering starting such a clinic and the rationale for that decision. They were also asked whether they had previously supported a resident continuity clinic but discontinued it within the past 2 years; if the response were positive, the reasoning behind the decision was elicited.

The first two survey questions helped categorize the clinics according to the level of supervision afforded the residents (Additional file 1). Specifically, we asked whether a resident-hosted clinic was supported by a training program, and if so, the degree of interaction required by the supervisors. If there were a resident-hosted clinic, respondents were asked whether the faculty supervisor were required to see every resident-treated patient. If not, then the respondents were asked whether the supervisor must discuss every case with the resident (but not necessarily see that patient). Four categories arose from this approach; they are listed from lowest to highest with respect to the degree of resident autonomy:

1. No resident-hosted clinic
2. Resident hosted clinic where the supervisor must see every resident patient
3. Resident hosted clinic where the supervisor must discuss every patient with the resident but not necessarily see the patient
4. Resident hosted clinic where the supervisor was not required to discuss every patient with the resident.

We assigned an ordinal score from 0 to 3 to these categories so that we might explore whether existed statistical correlations between the score and other surveyed variables. For the variable of number of residents in the program, we employed the Wilcoxon rank sum test using the median range of numbers of residents for programs falling into the four categories (0–3). For the variable of whether a program had 20 or few core faculty or whether a clinic was located in an urban demographic environment, we employed the Chi-squared statistic. For the variable of the contribution of the resident clinic to resident surgical volumes, we employed the Fisher exact test.

Finally, we also explored whether there might be correlations between other survey metrics such as the median number of residents against the number of faculty or against the contribution of the resident clinics to resident surgical volume (Wilcoxon rank sum test); median rather than mean number of residents was used because the number of residents is not normally distributed.

## Results

Responses were collected from 108 (94%) of the 115 ACGME accredited ophthalmology training programs. Of those respondents, 67 program directors (58% of total programs) expressed a desire to partner in this and future efforts, thereby creating the Ophthalmology Program Directors’ Study Group (OPDSG). Of the 108 respondents, 74 (69%) indicated that their trainees hosted continuity clinics while 34 (31%) did not. Furthermore, of those 74 programs hosting a continuity clinic, a spectrum of faculty oversight was demonstrated; 22 (30%) required a faculty member to see each patient treated by a resident, while 21 (28%) did not have such a requirement. However, 31 (42%) of programs required a faculty member to discuss every patient with the resident but not necessarily see the patient.

For those 22 programs hosting a resident clinic with the highest degree of supervision, (i.e., in which a supervisor saw and discussed every patient with the resident), 73–91% indicated that they reason for this practice was to maximize teaching, ensure compliance, provide the highest quality of care and/or comply with billing requirements at that institution. Only 54% did this to maximize clinic financial margins.

It was presumed that the 22 programs requiring a faculty member to see and discuss every patient with the resident also required that faculty member to document this discussion in the medical record. Of the 52 remaining programs with resident continuity clinics, 40 (77%) required the patient’s medical record to be reviewed and signed by a faculty member, regardless of whether the faculty member saw the patient. Reasons for this practice included maximizing opportunity for didactic feedback (69%), compliance with documentation standards of the program (95%), and ensuring highest quality documentation (69%)). Combining the 22 programs where faculty see every resident-treated patient and the 40 programs where charts are reviewed regardless of whether a patient is seen by the attending, one can calculate that 84% (62/74) of training programs with a resident continuity clinic expect resident charts to be reviewed and countersigned. Concerning the remaining 2 programs where chart sign-off was not generally done, at least ½ of ﻿the charts required sign-off if the attending ultimately saw the patient, the patient were a child, or if a surgical procedure were planned.

Concerning the physical presence of a faculty member, 72 (97%) programs assigned supervisors to their resident continuity clinics. These faculty members were on-site in the clinic in 66 (92%) programs, or stationed nearby in the remainder. Indeed, those faculty proved to be busy even in those 21 clinics with the most resident autonomy (i.e., an attending was not required to see or discuss every case with the resident). Of those programs, more than 2/3 of respondents indicated that the supervising faculty member would be expected to discuss the case at the resident’s request, and/or depending upon the level of training of the resident, or if a procedure (in-office or in the operating room) were planned.

As for the demographic setting of the training programs, multiple choices were permitted; more than half of all programs, regardless of whether they hosted a resident clinic, were reported to be in an urban setting. In addition more than 40% of either type of program provided care to underserved patients and in public hospitals. Notably, there were no statistically significant differences between programs with and without a resident continuity clinic in terms of demographic setting. Table 1 provides a summary of these demographic indices as well as the average numbers of residents hosted by the programs.

**Table 1:** Summary of Demographic Settings and Averaged Numbers of Resident-trainees for Programs Responding to Survey. The percentage (%) and number (n) of programs located in various demographic settings (Column 1) are presented. In addition, the averaged numbers of residents for programs is presented

	All programs	Programs with a resident-hosted continuity clinic	Programs without a resident-hosted continuity clinic
Training Program Demographic Setting			
Urban - % (n)	69% (75)	76% (55)	59% (20)
Suburban - % (n)	19% (21)	18% (13)	24% (8)
Veteran - % (n)	40% (43)	35% (26)	50% (17)
Rural - % (n)	6% (6)	5% (4)	6% (2)
Underserved - % (n)	43% (46)	45% (32)	41% (14)
Native American - % (n)	3% (3)	3% (2)	3% (1)
Public Hospital -% (n)	44% (47)	41% (30)	50% (17)
Private Hospital - % (n)	24% (26)	24% (17)	27% (9)
Referral Center - % (n)	41% (44)	47% (34)	29% (10)
Averaged Number of Resident-Trainees	13 (+/−5)	13 (+/−5)	13 (+/− 4)

Concerning the contribution the resident continuity clinics made to the resident surgical volumes, 54 (73%) of the 74 programs tracked this metric. Of those 54 programs, 36 (67%) reported that the resident outpatient clinics provided more than half of the resident surgical volumes. However, there was no statistically significant correlation between resident surgical volume contribution of the clinic and the number of residents in a training program (*p* = 0.21). On the other hand, there was a statistically significant difference between contribution to resident surgical volume and whether a program clinic provided care to veterans (*p* = 0.02), in that residents in the majority of programs with this type of clinic obtained fewer than 81% of their surgeries from this setting. No other demographic location (urban, referral, rural, underserved, private, public) demonstrated significant differences regarding contribution to resident surgical volume of a resident clinic.

Concerning faculty, we did find a statistically significant (*p* < 0.0001) and positive correlation between the number of resident trainees in a program and the number of core faculty. For the programs with 0–10 core faculty, the median number of residents was 10. For programs with 11–20, 21–30 and more than 31 core faculty, the mean number was 12, 15 and 18 respectively. On the other hand, there was no significant correlation between the number of residents and the number of full time faculty (*p* = 0.09). Table 2 provides the number of faculty for those programs with and without a continuity clinic.

**Table 2:** Sizes of the faculty populations supporting the ophthalmology-resident training programs responding to the survey. The percentage (%) and number (n) of programs with the population of Core, Full- and Part-Time faculty falling within the numerical range shown in Column 1 are presented

Programs with a resident-hosted continuity clinic				Programs without a resident-hosted continuity clinic		
Range for population numbers of faculty members	Core	Full Time	Part Time	Core	Full Time	Part Time
0–10	37% (27)	27% (20)	71% (52)	35% (12)	21% (7)	65% (22)
11–20	38% (28)	38% (28)	10% (7)	38% (13)	38% (13)	32% (11)
21–30	15% (11)	18% (13)	7% (5)	24% (8)	30% (10)	0%
31–40	7% (5)	8% (6)	6% (4)	3% (1)	3% (1)	0%
41–50	3% (2)	3% (2)	4% (3)	0%	3% (1)	3% (1)
51–60	1% (1)	1% (1)	0%	0%	3% (1)	0%
61–70	0%	0%	0%	0%	3% (1)	0%
71–80	0%	1% (1)	0%	0%	0%	0%
81–90	0%	0%	0%	0%	0%	0%
91–100	0%	1% (1)	1% (1)	0%	0%	0%
Greater than 100	0%	1% (1)	1% (1)	0%	0%	0%

No significant correlations were found between the different categories of clinics and the number of residents in a program (Table 1), the demographic site of the resident clinic (urban, suburban, rural, veteran, underserved, public, private, referral center), number of faculty and the contribution of the resident-hosted clinics to overall resident surgery volumes.

Of the 34 programs without resident continuity clinics, 4 (12%) discontinued this clinic setting in the past 2 years; the reasons for this were compliance and training concerns or insufficient faculty engagement. Notably, 2 (3%) of the 74 programs with clinics planned upon discontinuing this environment in the next 2 years, and both programs indicating that the clinics did not provide a sufficiently strong learning opportunity. On the other hand, 12 (35%) programs were either planning or considering starting this teaching environment in the next 2 years and 75% or more of those clinics cited the desire for increased resident autonomy and/or the perceived educational value of a longitudinal experience in which residents have ownership of patients.

Finally, 66 respondents (58%) indicated a willingness to join into a newly formed Ophthalmology Program Directors’ Study Group (OPDSG), to further explore the data collected by this study and propose further efforts of this type.

## Discussion

To the authors’ knowledge, this study is the first of its kind in exploring the landscape of ophthalmology resident outpatient clinics with respect to supervision and, by extrapolation, autonomy. An important strength of this study is the high response rate to the survey; although we were hoping for a reasonable sample, we acquired a nearly complete view of programs in the United States. On the other hand, it is possible that the responses from the remaining 6% of programs could have altered the statistical analyses. Another potential weakness in this study was that some questions may have been lacking clarity. For example, we assumed that respondents would be familiar with terms such as “core, full- and part-time faculty” but it is possible that providing the ACGME’s explicit definition of these terms [23] would have provided more accurate answers. In addition, the authors did not take into account the contribution made by volunteer faculty; a 2000 study of internal medicine residency training programs suggest that unpaid faculty provide a significant teaching contribution [24]. It must be recognized that this study is a snapshot and the results clearly indicating that the landscape of resident education is fluid. Therefore, the validity of this study could be reduced over time unless the study is repeated. A final caveat is that socially desirable responses to the survey questions might be encouraged by the current ACGME policies and/or the invitation to join the OPDSG. Such responses might skew the data in an unpredictable way since the ACGME fosters a climate of both enhanced supervision and resident autonomy.

A potential criticism of this study stems from its very premise, i.e., that supervision and autonomy are diametrically opposed. In fairness, this may not be entirely correct. A number of models have been published suggesting that supervision can be enhanced without severely compromising trainee autonomy [9, 19, 25, 26]. While the authors believe that this study reasonably measures important of aspects of supervision in the outpatient clinic setting, they concede that autonomy is a perception [14, 27, 28] and may be much more difficult to measure. However, the significant body of published literature describing the tension between autonomy and supervision suggest that they most likely vary inversely [15, 29–32].

Our results might also provide a benchmark for the degree of supervision provided by any particular program; this would permit each program to compare their category with the resident perception of supervision queried in the surveys distributed annually by the ACGME [33]. Furthermore, it would be reasonable to compare autonomy to metrics of clinical efficiency with an effort to better understand how supervision impacts clinic flow [34, 35]. In addition, a better understanding of the training environment in the United States may be useful adjunct toward current efforts to standardize training internationally by the International Council of Ophthalmology [36]. Recent reports indicate that there is significant variability among residents in non-US training programs with the degree of supervision and exposure to surgical experience they are offered [37–39].

Concerning the categories of clinics based upon autonomy, the authors concede that they might not have taken into account all necessary factors related to autonomy. The categories were based primarily upon the processes dictating interaction between the resident and supervising faculty in the resident-hosted clinic. It may prove useful in further studies to expand the variables employed so that more granular categories could be created. For example, these might include the detailed circumstances under which an attending might be required to discuss or see a patient if they were not otherwise mandated to do so. Notably, the authors did not include the variable of whether faculty were mandated to review the medical records of every patient seen by residents once the survey results made it clear that the majority of programs with resident continuity clinics setting already have this requirement. The survey results also made it clear that this mandated review of documentation added a layer of supervision without impeding clinic flow, i.e., the charts were reviewed *post facto*. Recognizing that the interactions between faculty members and ophthalmology residents in the outpatient setting have been reported to average almost 6 min per patient seen by the resident [34], mandated review by faculty of patient charts where the resident was the only provider may prove an increasingly helpful adjunct toward providing supervision without compromising autonomy. Disadvantages of this approach include limiting real-time feedback to trainees as well as the risk of finding problems after the fact rather than in real-time, making it more difficult to correct potential defects in the patient care provided by the resident. In addition, thorough chart reviews require a great deal of time spent after clinic hours and this could be extremely burdensome on faculty.

A previous series of papers described an important effort to create and validate a “resident supervision index” in 2010 [40–42]. This supervision index was dependent upon the amount of time (in minutes) a faculty member spent with a trainee. The categories used to define supervision/autonomy in the current study probably correlate directly with time required for faculty-trainee interaction and it would be interesting to measure the length of interactions for the different supervision styles we explored. This could provide insight into the impact of supervision styles on clinic flow. It could also help guide the creation of resident scheduling templates to ensure sufficient learning opportunities while avoiding delays in patient care.

The authors believe that the categorization of resident clinics based upon supervision and autonomy has the potential to be more than an academic exercise, but this will require validation to some other accepted and established metrics. However, at present, there are no published data of this type. Incomplete rankings for graduate medical education programs have been popularized but recent publications indicate that their dependence upon reputation data rather than true measures of quality vitiate their usefulness [43–45]. On the other hand, research productivity certainly could be considered a useful metric [46] and data such as grant funding through the NIH [47] and number of publications in a given year (gleaned through online bibliographic resources) are relatively accessible. That being said, research prowess does not necessarily translate into skillful clinical decision making or teaching ability.

Certain metrics are regularly collected by residency training programs and, if made readily available, would permit the ranking of programs. Examples of these include resident surgical case volume, scores on resident in-service examinations, first-time pass rate of board examinations, results of fellowship and residency matches, and annual surveys of residents and of faculty conducted by the ACGME. While it is unlikely that individual residency programs or the ACGME would share these data publicly, these sort of rankings would be reasonable to help us determine whether the categorization system we created based upon resident supervision and autonomy might correlate with other objective measures of residency programs.

Recently, the Wilmer Eye Institute published data concerning the contribution of its resident continuity clinic to resident surgical volume [48]. The present study provides a snapshot of this metric for most of the ophthalmology programs in the United States. It appears that continuity clinics provide most residents the majority of their surgical experiences, indicating that these learning environments enhance both clinical and surgical education. It seems reasonable to suggest that most programs believe resident continuity clinics provide significant value, since more than 2/3 of programs in the United States have such a clinic. Furthermore, 12 programs without a resident continuity clinic were considering developing one, which is twice the number of programs that either recently discontinued one or were considering doing so. The fact that the resident-hosted clinics provide the majority of resident surgical cases in their respective programs may also be a strong incentive to maintain or create this learning environment. Notably, the data seem to support the idea that while a resident clinic located at a facility caring for veterans will likely provide a valuable source of surgical education opportunities, residents can expect to glean fewer than 4/5 of their cases from this setting.

Concerning further work in this area, the OPDSG’s first priority may be to repeat this survey over regular intervals, since the results make it clear that programs plan to discontinue or start continuity clinics within the next 2 year. The results from this study could also be used to explore whether a program’s approach to autonomy and supervision in the outpatient setting is a factor that might be considered by candidates applying for an ophthalmology training position or in recruiting teaching faculty. Although recent publications suggest that faculty supervision and mentoring are important to applicants, the respondents were not applicants to ophthalmology training programs [49, 50]. The authors could find no published studies of factors influencing medical students to rank residency programs in which this variable was specifically queried.

In follow up surveys, more granular data might be useful. Most programs indicated that a faculty member would review the resident’s examination and/or documentation based upon the level of resident training, suggesting that good compliance with the ACGME’s expectation of graduated autonomy. However, specifically querying how this is structured, e.g., by milestones, resident seniority, etc., could be informative. Additionally, it could be useful to explore how autonomy and supervision are provided in other settings, such as the Emergency Department or night-call. Future surveys might also specifically explore the perceived value and need of resident continuity clinics. We queried programs about their rationale for choosing to adopt or cancel these clinics, but we might want to open this question to all programs currently supporting a clinic, thereby understanding why they want to maintain it. Finally, it might prove informative to survey young ophthalmologists across the country about how prepared they felt entering practice to determine whether that might correlate to the degree of autonomy provided by their training environment.

Identifying measures of quality is mission-critical to resident education. Validated tools are not readily available yet clearly needed. In order to develop best practices for teaching, residency training programs require benchmarks. Future studies are needed to determine whether the categorization of clinics based upon supervision and autonomy will be useful.

## Conclusions

This study has found that the majority of ACGME-accredited ophthalmology resident training programs in the United States host a continuity clinic where residents follow their own patients. Furthermore, most of these clinics require supervising faculty to review both the patients seen and the medical documentation created by the resident encounters. In addition these faculty are assigned to the clinic providing on-site access. Furthermore, while the number of core faculty correlates positively with the number of residents in a program, there is no correlation between the number of core faculty and the degree of supervision in the resident-hosted clinics. The resident continuity clinic appears to provide the majority of residents with the majority of their surgical cases. The majority of continuity clinics serve an urban population and a large minority are dedicated to providing care to the underserved.

The categorization of resident clinics based upon degree of supervision and autonomy might provide a new perspective to help establish best practices for an ophthalmology training program, presuming it can be correlated with other such metrics. We might also use this system as a benchmark for the degree of supervision provided by a training program; this would permit a program to compare itself with the resident perception of supervision queried in the survey distributed annually by the ACGME. Finally, mapping the training landscape in the United States could be useful to the international effort to standardize ophthalmology resident education.

## Additional file

Additional file 1: Resident Clinic Survey. (PDF 176 kb)

## Abbreviations

ACGME: Accreditation Council For Graduate Medical Education
IRB: Institutional Review Board
OPDSG: Ophthalmology Program Director’s Study Group
