# Supplementary material for: Supervision and autonomy of ophthalmology residents in the outpatient Clinic in the United States: a survey of ACGME-accredited programs
Source: BMC Med Educ. 2017 Jun 26;17:105. doi: 10.1186/s12909-017-0941-0 (PMC5485577; doi:10.1186/s12909-017-0941-0)
Supplement: Additional file 1: — Resident Clinic Survey. (PDF 176 kb) [file 12909_2017_941_MOESM1_ESM.pdf]

Appendix 1: Resident Clinic Survey

Q1

Does your program provide an outpatient clinic where residents provide continuity care to their own comprehensive ophthalmology patients with or without direct supervision?

NOTE: If your program has more than 1 clinic where residents provide continuity care to their own comprehensive patients, please have in mind the clinic at the PRIMARY INSTITUTION of your program or at the clinic where the residents see the majority of their own comprehensive patients.

If you answer NO you will jump to Question 12.

- ◯ Yes
- ◯ No

Q2

Must a faculty member discuss every patient treated by a resident with that resident?

If you answer Yes-but that patient might not necessarily be seen the faculty member, you will jump to Question 5
If you answer YES you will jump to Question 4
If you answer NO you will jump to Question 3

- ◯ YES and that patient must also be seen by the faculty member who will co-sign the documentation of the encounter
- ◯ YES but that patient might not necessarily be seen by the faculty member
- ◯ NO

Q3

Which of the following circumstances would require a faculty member to discuss with the resident the care offered by the resident (but not necessarily see the patient)?
PLEASE CHECK ALL THAT APPLY.
When finished please click on ***COMPLETE*** response at the end of the list and then advance.
You will be directed to ***Question 5***

- ☐ A resident requests input by the faculty member
- ☐ New patient to the clinic
- ☐ Established patient with a new diagnosis
- ☐ Pediatric patient

- ☐ The level of training of the resident
- ☐ Other resident-dependent criteria such as progress reaching certain milestones
- ☐ Patient for whom there is a decision to offer surgery in the operating room
- ☐ Patient for whom there is a decision to offer an in-office procedure (e.g., laser, punctal occlusion, intra-vitreal injection)
- ☐ Patient seen early in the academic year
- ☐ Patient not seen by a faculty member previously
- ☐ Patient not seen by a faculty member within the past year
- ☐ Patient not seen by a faculty member within the past 2 years
- ☐ A diagnosis that requires initiation of new therapy (e.g., uncontrolled glaucoma, uncontrolled uveitis)
- ☐ A diagnosis that requires initiation of systemic therapy
- ☐ A diagnosis that requires consultation to a discipline outside ophthalmology
- ☐ A diagnosis that requires in-office imaging (e.g., OCT, B-scan)
- ☐ A diagnosis that requires laboratory testing
- ☐ A diagnosis that requires radiologic imaging
- ☐ When there is a need to obtain formal informed consent
- ☐ Other
- ☐ ***COMPLETE***

Q4

What are the reasons that faculty members are required to see every patient treated by a resident and sign the documentation of every resident-patient encounter?

PLEASE CHECK ALL THAT APPLY.

When finished please click on ***COMPLETE*** response at the end of the list and then advance. You will be directed to ***Question 9.***

- ☐ Maximize opportunities to teach residents
- ☐ Compliance with program policies on resident supervision
- ☐ Ensure that the highest quality of care is delivered
- ☐ Billing (e.g., insurer/payor) requires faculty sign-off
- ☐ Ensure financial viability of clinic or productivity of faculty supervising residents
- ☐ Other
- ☐ ***COMPLETE***

Q5

Which of the following circumstances would require a faculty member ***to see*** a patient treated by a resident and therefore co-sign the chart for that encounter (***please check all that apply)***

You will go to Question 6 next.

- ☐ A resident requests input by the faculty member
- ☐ New patient to the clinic
- ☐ Established patient with a new diagnosis
- ☐ Pediatric patient
- ☐ The level of training of the resident
- ☐ Other resident-dependent criteria such as progress reaching certain milestones
- ☐ Patient for whom there is a decision to offer surgery in the operating room
- ☐ Patient for whom there is a decision to offer an in-office procedure (e.g., laser, punctal occlusion, intra-vitreal injection)
- ☐ Patient seen early in the academic year
- ☐ Patient not seen by a faculty member previously
- ☐ Patient not seen by a faculty member within the past year
- ☐ Patient not seen by a faculty member within the past 2 years
- ☐ A diagnosis that requires initiation of new therapy (e.g., uncontrolled glaucoma, uncontrolled uveitis)
- ☐ A diagnosis that requires initiation of systemic therapy
- ☐ A diagnosis that requires consultation to a discipline outside ophthalmology
- ☐ A diagnosis that requires in-office imaging (e.g., OCT, B-scan)
- ☐ A diagnosis that requires laboratory testing
- ☐ A diagnosis that requires radiologic imaging
- ☐ When there is a need to obtain formal informed consent
- ☐ Other:

Q6

Must a faculty member co-sign the documentation of every resident-patient encounter ***regardless*** of whether the patient was seen by or discussed with the faculty member?

If you answered YES you will go to Question 7
If you answered NO you will skip to Question 8

- ○ Yes
- ○ No

Q7

What are the reason(s) that faculty members are required to co-sign the documentation of every resident-patient encounter ***regardless*** of whether the patient was seen by or discussed with the faculty member?

PLEASE CHECK ALL THAT APPLY

When finished please click on ***COMPLETE*** response at the end of the list and then advance. You will be directed to ***Question 9.***

- ☐ Maximize opportunities to provide feedback on documentation to residents
- ☐ Compliance with program policies on resident supervision
- ☐ Ensure that the highest quality of documentation is achieved
- ☐ Other
- ☐ ***COMPLETE***

Q8

Which of the following circumstances would require that a resident have a chart co-signed by a faculty member? (***please check all that apply)***

You will go to Question 9 next

- ☐ The faculty member must sign-off if they examine the patient
- ☐ New patient to the clinic
- ☐ Established patient with a new diagnosis
- ☐ Pediatric patient
- ☐ The level of training of the resident
- ☐ Other resident-dependent criteria such as progress reaching certain milestones
- ☐ Patient for whom there is a decision to offer surgery in the operating room
- ☐ Patient for whom there is a decision to offer an in-office procedure (e.g., laser, punctal occlusion, intra-vitreal injection)
- ☐ Patient seen early in the academic year
- ☐ Patient not seen by a faculty member previously
- ☐ Patient not seen by a faculty member within the past year
- ☐ Patient not seen by a faculty member within the past 2 years
- ☐ A diagnosis that requires initiation of new therapy (e.g., uncontrolled glaucoma, uncontrolled uveitis)
- ☐ A diagnosis that requires initiation of systemic therapy
- ☐ A diagnosis that requires consultation to a discipline outside ophthalmology
- ☐ A diagnosis that requires in-office imaging (e.g., OCT, B-scan)
- ☐ A diagnosis that requires laboratory testing
- ☐ A diagnosis that requires radiologic imaging
- ☐ When there is a need to obtain formal informed consent

- ☐ Other: ______

Q9

Is there a specific faculty member ***assigned to be available*** to the resident-hosted clinic for every session?

If YES you will go to Question 10
If NO you will go to Question 11

- ○ Yes
- ○ No

Q10

Does the faculty member assigned to the resident-hosted clinic provide ***on-site*** supervision by being stationed within the resident clinic?

If YES you will go to Question 16
If NO you will skip to Question 11

- ○ Yes
- ○ No
- ○ Sometimes

Q11

Please describe where a resident might turn if he/she needs to ask a faculty member a question about a patient seen in the resident clinic
PLEASE CHECK ALL THAT APPLY

When finished please click on ***COMPLETE*** response at the end of the list and then advance. You will be directed to ***Q16.***

- ☐ Contact a supervising attending stationed near the clinic
- ☐ Leave the clinic to speak to an attending elsewhere in the building
- ☐ Telephone/text message a faculty member
- ☐ Other: ______
- ☐ ***COMPLETE***

Q12

Did your program discontinue an outpatient clinic where residents saw their own patients within the past 2 years?

If YES you will go to Question 13
If NO you will skip to Question 14

- Yes
- No

Q13

Please explain why you discontinued the clinic where residents see their own patients.
PLEASE CHECK ALL THAT APPLY

When finished please click on ***COMPLETE*** response at the end of the list and then advance. You will be directed to ***Question 16.***

- Compliance reasons
- Billing Concerns
- Training Concerns
- Expenses of running the clinic
- Insufficient faculty engagement or commitment
- Other
- ***COMPLETE***

Q14

Do you have any plans to start a clinic where residents see their own patients within the next 2 years?

If YES you will go to Question 15
If NO you will skip to Question 20

- Yes
- No
- Maybe

Q15

Please provide the rationale behind your response to Q14.
PLEASE CHECK ALL THAT APPLY

When finished please click on ***COMPLETE*** response at the end of the list and then advance. You will be directed to ***Question 20.***

- Desire to increase resident autonomy
- Create a location where almost any patient can be assured to get an appointment
- Increase rapidity with which patients can get an appointment
- Educational value of a longitudinal experience in which residents have ownership of patients

- ☐ Other: ______
- ☐ ***COMPLETE***

Q16

Have you tracked the contribution to resident surgical experiences provided by the clinic hosted by your residents?

If YES you will go to Question 17
If NO you will skip to Question 18

- Yes
- No

Q17

What percentage of the overall resident surgical experience (AS PRIMARY SURGEON) came from the resident clinic?

- 0-10%
- 11-20%
- 21-30%
- 31-40%
- 41-50%
- 51-60%
- 61-70%
- 71-80%
- 81-90%
- 91-100%

Q18

Do you have plans to discontinue your resident-hosted clinic within the next 2 years? (If you already discontinued your resident clinic, please click "Not Applicable"

If YES you will go to Question 19
If NO you will skip to Question 20
If NOT APPLICABLE you will skip to Question 20

- Yes
- No
- Not Applicable; the resident clinic has been discontinued

Q19

Please explain the rationale behind your answer to Q18 (***please check all that apply)***

You will go to Question 20 next

- ☐ Compliance reasons
- ☐ Billing Concerns
- ☐ Training Concerns
- ☐ Expenses of running the clinic
- ☐ Other

Q20

Please indicate the HEALTHCARE SETTING that best describes the clinic environment in which your residents serve (***please check all that apply)***

You will go to Questions 21 next

- ☐ URBAN
- ☐ SUBURBAN
- ☐ RURAL
- ☐ VETERAN
- ☐ UNDERSERVED
- ☐ AMERICAN INDIAN
- ☐ PUBLIC HOSPITAL
- ☐ PRIVATE HOSPITAL
- ☐ REFERRAL CENTER

Q21

Please use the slider to indicate the total number of residents in your program:

	03	6	9	12	15	18	21	24	27	30
Number of residents in program										

Q22

Please use the slider below to indicate how many core, part-time and full-time faculty members are in your residency program:

	0-10	11-20	21-30	31-40	41-50	51-60	61-70	71-80	81-90	91-100	101-110	111-120	121-130	131-140	141-150
Core faculty:	☐	☐	☐	☐	☐	☐	☐	☐	☐	☐	☐	☐	☐	☐	☐
Full time faculty:	☐	☐	☐	☐	☐	☐	☐	☐	☐	☐	☐	☐	☐	☐	☐

	0-10	11-20	21-30	31-40	41-50	51-60	61-70	71-80	81-90	91-100	101-110	111-120	121-130	131-140	141-150
Part-time faculty	☐	☐	☐	☐	☐	☐	☐	☐	☐	☐	☐	☐	☐	☐	☐

Q23

Please click on the arrow in the dropdown box, then find and click on the tab with your residency program. If it is not on the list, please free text the name of your program in the "Add a Comment" below this list.

Q24

Please provide your name, email address and name of residency program.

We welcome you to partner with us in future surveys exploring factors that impact resident education.

If you would like to share in this effort and join our AUPO Resident Clinic Study Group, please click on the "Count Me In" tab.

- ☐ Name
- ☐ email
- ☐ Count Me In
